# Antigenic Characterization of Circulating and Emerging SARS-CoV-2 Variants in the U.S. throughout the Delta to Omicron Waves

**DOI:** 10.3390/vaccines12050505

**Published:** 2024-05-07

**Authors:** Han Di, Elizabeth A. Pusch, Joyce Jones, Nicholas A. Kovacs, Norman Hassell, Mili Sheth, Kelly Sabrina Lynn, Matthew W. Keller, Malania M. Wilson, Lisa M. Keong, Dan Cui, So Hee Park, Reina Chau, Kristine A. Lacek, Jimma D. Liddell, Marie K. Kirby, Genyan Yang, Monique Johnson, Sharmi Thor, Natosha Zanders, Chenchen Feng, Diya Surie, Jennifer DeCuir, Sandra N. Lester, Lydia Atherton, Heather Hicks, Azaibi Tamin, Jennifer L. Harcourt, Melissa M. Coughlin, Wesley H. Self, Jillian P. Rhoads, Kevin W. Gibbs, David N. Hager, Nathan I. Shapiro, Matthew C. Exline, Adam S. Lauring, Benjamin Rambo-Martin, Clinton R. Paden, Rebecca J. Kondor, Justin S. Lee, John R. Barnes, Natalie J. Thornburg, Bin Zhou, David E. Wentworth, Charles Todd Davis

**Affiliations:** 1 Influenza Division, Centers for Disease Control and Prevention, Atlanta, GA 30329, USA; 2 Division of Core Laboratory Services and Response, Centers for Disease Control and Prevention, Atlanta, GA 30329, USA; 3 Coronavirus and Other Respiratory Viruses Division, Centers for Disease Control and Prevention, Atlanta, GA 30329, USA; 4 Eagle Global Scientific, Inc., Atlanta, GA 30341, USA; 5 Synergy America, Inc., Duluth, GA 30329, USA; 6 Vanderbilt Institute for Clinical & Translational Research, Vanderbilt University Medical Center, Nashville, TN 37232, USA; 7 Department of Medicine, Wake Forest School of Medicine, Winston-Salem, NC 27101, USA; 8 Department of Medicine, Johns Hopkins University School of Medicine, Baltimore, MD 21205, USA; 9 Department of Emergency Medicine, Beth Israel Deaconess Medical Center, Boston, MA 02215, USA; 10 Department of Medicine, The Ohio State University, Columbus, OH 43210, USA; 11 Departments of Internal Medicine and Microbiology and Immunology, University of Michigan, Ann Arbor, MI 48109, USA

**Keywords:** SARS-CoV-2, COVID-19 vaccine, Delta variant, Omicron variant, antigenic characterization, neutralizing antibody

## Abstract

Severe acute respiratory syndrome coronavirus 2 (SARS-CoV-2) has evolved into numerous lineages with unique spike mutations and caused multiple epidemics domestically and globally. Although COVID-19 vaccines are available, new variants with the capacity for immune evasion continue to emerge. To understand and characterize the evolution of circulating SARS-CoV-2 variants in the U.S., the Centers for Disease Control and Prevention (CDC) initiated the National SARS-CoV-2 Strain Surveillance (NS3) program and has received thousands of SARS-CoV-2 clinical specimens from across the nation as part of a genotype to phenotype characterization process. Focus reduction neutralization with various antisera was used to antigenically characterize 143 SARS-CoV-2 Delta, Mu and Omicron subvariants from selected clinical specimens received between May 2021 and February 2023, representing a total of 59 unique spike protein sequences. BA.4/5 subvariants BU.1, BQ.1.1, CR.1.1, CQ.2 and BA.4/5 + D420N + K444T; BA.2.75 subvariants BM.4.1.1, BA.2.75.2, CV.1; and recombinant Omicron variants XBF, XBB.1, XBB.1.5 showed the greatest escape from neutralizing antibodies when analyzed against post third-dose original monovalent vaccinee sera. Post fourth-dose bivalent vaccinee sera provided better protection against those subvariants, but substantial reductions in neutralization titers were still observed, especially among BA.4/5 subvariants with both an N-terminal domain (NTD) deletion and receptor binding domain (RBD) substitutions K444M + N460K and recombinant Omicron variants. This analysis demonstrated a framework for long-term systematic genotype to antigenic characterization of circulating and emerging SARS-CoV-2 variants in the U.S., which is critical to assessing their potential impact on the effectiveness of current vaccines and antigen recommendations for future updates.

## 1. Introduction 

**Figure 1:**
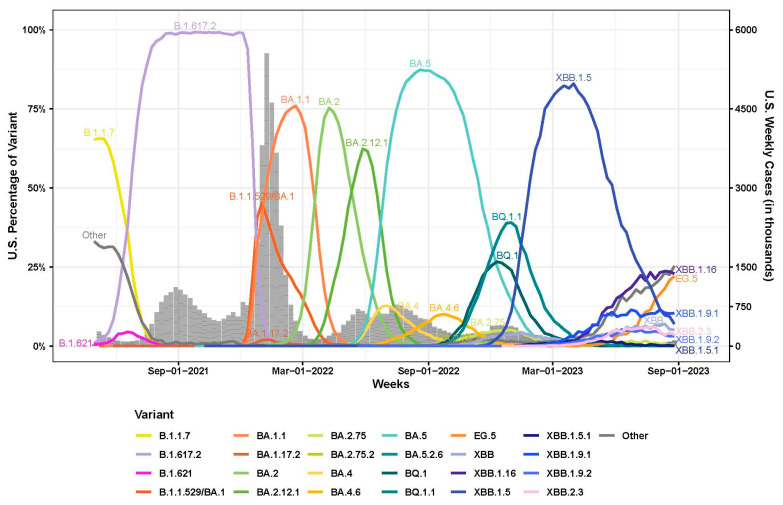
Weekly proportions of SARS-CoV-2 variants in the U.S. with underlying U.S. case counts (1 May 2021 to 1 September 2023). Percentage of SARS-CoV-2 variants in the U.S. (line graph, 0% to 100%) were assembled from all available U.S. sequencing surveillance data. Variants represent an aggregation of many individual pangolin lineages aliased under a major parental lineage designation and were labeled with different colors. U.S. Weekly case counts were obtained from HHS Protect and are summarized in the grey bar graph (right-side, dual Y-axis).

Over the course of the COVID-19 pandemic, SARS-CoV-2 has caused multiple epidemics waves in the United States of America (U.S.) [1,2,3,4,5]. The Delta wave in the U.S. started in the summer of 2021 and was mainly driven by B.1.617.2 and its subvariants [5,6,7] (Figure 1). The Omicron variants (B.1.1.529), which carried more than 30 mutations in the spike protein compared to the Wuhan-Hu-1 progenitor, was first reported in November 2021 [8]. Since then, Omicron has evolved into numerous subvariants with additional spike amino acid substitutions and deletions that exhibited enhanced immune evasion. By January 2022, the Omicron wave replaced the Delta wave and was predominated by BA.1 and BA.1.1 first, then BA.2 and BA.2.12.1, followed by BA.5, BQ.1/BQ.1.1 and XBB.1.5 as of September 2023 [9,10,11,12] (Figure 1). 

In December 2020, the first COVID-19 mRNA vaccines were authorized in the U.S. under emergency use authorization (EUA). The Janssen viral vector COVID-19 vaccine received an EUA from FDA in February 2021 but is no longer available for use in the United States as of May 2023. In September 2021, the COVID-19 mRNA booster vaccine was authorized in the U.S. under EUA to be administered at least six months after a two dose primary series in eligible populations. Both the primary series and the booster mRNA vaccine were designed as a monovalent vaccine based on the original strain of SARS-CoV-2. With the emergence of divergent Omicron variants, bivalent formulations of the COVID-19 mRNA vaccines were recommended and authorized for use as a single booster dose in the U.S. under EUA in August 2022 [13]. The bivalent vaccines contained two mRNA components of SARS-CoV-2, one based on the Wuhan-Hu-1 spike sequence and the other based on the Omicron BA.4/BA.5 spike sequence. 

The spike protein on the surface of the SARS-CoV-2 virion is the main target of the human neutralizing antibodies elicited by infection or vaccination [14]. Human neutralizing antibodies that target the receptor binding domain (RBD) and N-terminal domain (NTD) of the spike protein contribute to the neutralizing activity of polyclonal antibodies against SARS-CoV-2 [15]. Antibodies that target the RBD can be categorized into four classes (class 1–4) based on structural analyses [16] or twelve epitope groups (group A-F3) based on the antibody escaping mutation profile [17,18]. Group A–D antibodies overlap with the class 1–2 antibodies and their epitopes overlap with RBD residues involved in ACE2 binding. Like class 1 antibodies, group A and B antibodies usually target RBDs in the “up” conformation. Similar to class 2 antibodies, most of the group C and D antibodies target RBDs regardless of their “up” or “down” conformations. Group E and F antibodies are similar to the class 3 and 4 antibodies, among which groups E1–E3 and F1 do not compete with ACE2. The antibodies that target the NTD can be classified into four classes: class α, β, γ and δ [19]. The class α antibodies target the well-known NTD antigenic supersite [20,21]. The epitopes of class β and δ could elicit relatively broad, but less potent, neutralizing antibodies. 

In November 2020, CDC initiated the National SARS-CoV-2 Strain Surveillance (NS3) program (https://www.aphl.org/programs/infectious_disease/SARS-CoV-2/Pages/Sequence-Based-Surveillance-Submission.aspx (accessed on 31 March 2024)) to monitor the evolution of circulating SARS-CoV-2 variants in the U.S. [4,10]. Through the NS3 program, CDC has received SARS-CoV-2 clinical specimens collected from across the nation. In this study, we isolated SARS-CoV-2 Delta, Mu and Omicron subvariants from selected clinical specimens received between May 2021 and February 2023 based on their spike sequences. To evaluate their antigenicity against vaccines previously authorized in the U.S., we performed systematic antigenic characterization on all isolated SARS-CoV-2 variants using vaccinee sera collected post primary series vaccination, post the third-dose original monovalent booster and post the fourth-dose bivalent booster. 

## 2. Materials and Methods 

### 2.1. Serum Samples

Vaccinee sera used in this analysis were received from the Investigating Respiratory Viruses in the Acutely Ill (IVY) Network, which was funded by the U.S. Centers for Disease Control and Prevention (CDC) to collect serum samples from volunteers to monitor the effectiveness of SARS-CoV-2 vaccines among U.S. adults [22]. Serum samples selected for this analysis were collected 12–56 days after last vaccination from volunteers who had no prior infection with SARS-CoV-2 based on negative anti-nucleocapsid antibodies detected in the serum samples. Three post-primary series vaccinee sera pools were generated as follows: (1) Moderna post-primary series pool from 10 individuals (median age: 23) who received 2 doses of the Moderna original mRNA monovalent vaccine (spike IgG: 605–8066 binding antibody units per mL (BAU/mL)). (2) Pfizer post-primary series pool from 10 individuals (median age: 32.5) who received 2 doses of the Pfizer-BioNTech original mRNA monovalent vaccine (spike IgG: 440–5227 BAU/mL). (3) Medium range post-primary series pool from 5 volunteers (median age: 49) who received 2 doses of the mRNA monovalent vaccine (Moderna or Pfizer-BioNTech) and had medium range spike-specific antibody titers (1339–3541 BAU/mL) in their serum samples (Supplementary Table S1). Twenty post-third-dose serum samples from volunteers (median age: 37) who received 3 doses of the original mRNA monovalent vaccine (Moderna or Pfizer-BioNTech) were used for this analysis (Supplementary Table S2), among which, 5 individual sera with medium range spike-specific antibody titers (159–3015 BAU/mL) were pooled together as a medium range post-third-dose pool (Supplementary Table S1). Eleven post-bivalent serum samples were also selected from volunteers (median age: 47.5) who received 3 doses of the original monovalent mRNA vaccine and 1 dose of the bivalent mRNA booster (original SARS-CoV-2 virus + Omicron variant BA.4/5) from Pfizer-BioNTech or Moderna (Supplementary Table S3). This activity was approved by each participating institution, either as a research project with written informed consent or as a public health surveillance project without written informed consent. This activity was also reviewed by the CDC and conducted in a manner consistent with applicable federal laws and CDC policies: see, e.g., 45 C.F.R. part 46.102(l)(2), 21 C.F.R. part 56; 42 U.S.C. §241(d); 5 U.S.C. §552a; 44 U.S.C. §3501 et seq. 

### 2.2. Biosafety Statement

All work involving infectious SARS-CoV-2 viruses was conducted in Biosafety Level 3 facilities with enhanced practices (BSL-3E) with institutional approval. All personnel working with the virus in the BSL-3E laboratories were trained with relevant safety protocols and relevant standard operating procedures (SOPs). To remove inactivated virus from BSL-3E for RNA purification and sequencing, virus inactivation and transfer protocols were established by the CDC’s Office of Laboratory Science and Safety. 

### 2.3. Prevalence Analysis of SARS-CoV-2 Variants

Proportions of SARS-CoV-2 variants were assembled from all available U.S. sequencing surveillance data from GISAID (https://gisaid.org/ (accessed on 26 January 2024)) and NCBI (https://www.ncbi.nlm.nih.gov/genbank/ (accessed on 26 January 2024)). U.S. weekly case counts were obtained from HHS Protect (https://www.cdc.gov/ncezid/hhs-protect/index.html (accessed on 26 January 2024)). Data available as of September 2023 were used. Variants represent an aggregation of many individual pangolin lineages aliased under a major parental lineage designation. Sequence Pangolin lineages were assigned using Pangolin PUSHER (PUSHER-v1.21, pangolin version 4.3.1, scorpio version 0.3.17, constellation version 0.1.12, usher version 0.6.2, pangolin data version 1.21, pangolin assignment version 1.21).

### 2.4. Virus Isolation from Clinical Specimens

SARS-CoV-2 clinical specimens (100 µL) were inoculated in confluent T-25 flasks containing either Vero/TMPRSS2 cells or Vero/TMPRSS2/ACE2 cells [23]. Inoculated flasks were incubated at 37 °C with 5% CO_2_ and observed daily up to 5 days post inoculation. Upon observation of cytopathic effect (CPE) and/or syncytia formation, culture media was collected and centrifuged at 2500 rpm for 5 min, and supernatant was aliquoted as passage 1 (P1) stocks and stored at −80 °C in a freezer. P1 stocks were then inoculated in confluent T-75 flasks containing Vero/TMPRSS2 cells at multiplicity of infection (MOI) of 0.01. Two days post inoculation, culture media were collected and centrifuged at 2500 rpm for 5 min. Supernatant was aliquoted as passage 2 (P2) stocks and stored at −80 °C freezer. 

### 2.5. Focus Reduction Neutralization Test (FRNT)

Each SARS-CoV-2 P2 virus analyzed was titrated and diluted to 4000–8000 focus forming units (FFUs) per ml in DMEM supplemented with 2% heat-inactivated fetal bovine serum (HyClone, Logan, UT, USA) and 1× Anti-Anti (Gibco). Human serum samples were heat-inactivated at 56 °C for 30 min, and serially diluted in 3-fold or 4-fold for 7 dilutions in sextuplicate in 96-well untreated round bottom plates. Serum dilution was started at 1:5 for post-primary series sera, 1:10 for post third-dose sera, or 1:20 for post-bivalent sera. Diluted sera were mixed with equal volume of diluted virus and incubated at room temperature for one hour. After removing the cell culture media from 96-well tissue culture plates containing confluent Vero/TMPRSS2 cells, cells were inoculated with 50 µL virus-sera mixture in each well. The plates were incubated at 37 °C with 5% CO_2_ for 1 h, overlaid with 100 µL 0.75% methylcellulose ((Sigma-Aldrich, St. Louis, MO, USA) in DMEM supplemented with 10% heat-inactivated fetal bovine serum (HyClone) and 1× Penicillin-Streptomycin (Gibco, Waltham, MA, USA), and then incubated at 33 °C with 5% CO_2_ for 20–22 h. The next day, methylcellulose was removed from each well and 100 µL 70% ethanol was added for 10 min to fix and permeabilize the cells. Cells were washed with PBS, blocked with SuperBlock Blocking Buffer (Thermo Scientific, Waltham, MA, USA) for 30 min at room temperature, and stained with SARS/SARS-CoV-2 Coronavirus Nucleocapsid Monoclonal Antibody (Thermo Scientific, MA5-29981) at 1:4000 dilution at 4 °C overnight. After washing with PBS, cells were stained with IgG (H + L) highly cross-adsorbed secondary antibody with Alexa Fluor™ Plus 647 (Thermo Scientific, A32728) at 1:400 dilution for 1 h at room temperature. All plates were imaged using the CellInsight CX5 High-Content Screening Platform (Thermo Scientific) under 2× magnification with the same setting. Fluorescent virus foci were identified and quantified using the Cellomics Scan Version 6.6.2 (Thermo Scientific, Build 8533) and exported in Excel and R for downstream analysis. FRNT_50_ values were calculated using a three-parameter log-logistic function (LL.3) in R. In cases where the hill constant was fit outside of the range from 0.5 to 2, a two-parameter fit while fixing the hill constant to 1 was used to estimate the FRNT_50_ values. The R script has been deposited in GitHub: https://github.com/CDCgov/SARS-CoV-2_FRNTcalculations/ (accessed on 15 January 2023).

### 2.6. Data Processing and Statistical Analysis

When using the pooled sera, FRNT_50_ neutralization titers of all the virus isolates with the same unique spike protein sequence (including FRNT_50_ values from all independent repeats) were used to calculate the geometric mean FRNT_50_ titer (GMT) for that unique spike protein. If there was only one virus isolate available for a particular spike protein sequence, then at least two independent FRNT assays were performed and neutralization titers of all independent repeats were used to calculate the GMT for that spike protein. When using the individual human serum samples, GMT for each virus was calculated using the FRNT_50_ neutralization titers of all the human serum samples analyzed against that virus. Each individual human serum sample was analyzed in at least two independent FRNT assays. For statistical analysis, FRNT_50_ neutralization titers were log transformed to better approximate the Gaussian distribution. Significance relative to the reference virus or ancestral virus was determined by one-way ANOVA with Dunnett’s multiple comparisons test for FRNT assays using the pooled sera, and one-way repeated measures ANOVA with Dunnett’s multiple comparisons test for FRNT assays using the individual human serum samples. *p* value was adjusted to account for multiple comparisons and the family-wise alpha threshold was set at 0.05. All graphs and statistical analysis were made in GraphPad Prism 9.

## 3. Results

### 3.1. Specimen Selection Process for Antigenic Characterization

**Figure 2:**
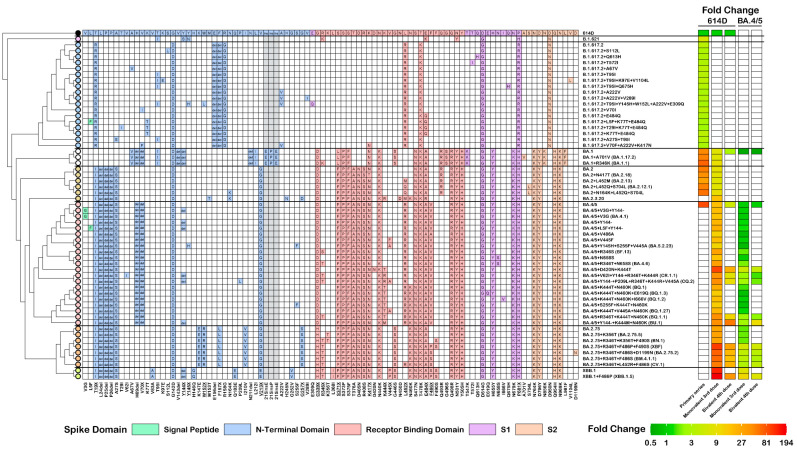
Genetic and antigenic summary of the analyzed SARS-CoV-2 Delta, Mu and Omicron variants. Spike mutations relative to the 614D reference virus (USA-WA1/2020) are shown with tables indicating the substitutions, insertions or deletions colored based on the spike protein domains. The reference amino acid at each position is labeled for the 614D reference at the top, and the amino acid changes are summarized at the bottom of the table. Residues with multiple different mutations are underlined. A dendrogram depicting the approximate relationships between analyzed variants has been added to the left of the mutation table. The variant names have been added to the right of the mutation table and are labeled by the parental virus with additional spike mutations relative to that parental virus. The antigenicity of all the analyzed variants are shown as neutralization fold change heatmaps colored from dark green, indicating a 0.5-fold change, to dark red, indicating a fold change of 194. Neutralization data using vaccinee sera post the primary series, monovalent third dose, and bivalent fourth dose are shown as fold change relative to 614D reference or the BA.4/5 variant.

Domestic SARS-CoV-2 clinical specimens received by the National SARS-CoV-2 Strain Surveillance (NS3) program were sequenced and analyzed to identify a wide range of emerging variants including those designated as variants of interest or variants of concern by the World Health Organization (WHO) and CDC. Additional specimens were selected if they met both of the following criteria: (1) specimens with spike mutations at residues linked to known or suspected functional or interaction sites and (2) specimens that were circulating in the U.S. at more than 0.5% prevalence or were quickly increasing in proportion. Specimens with spike mutations that were similar to the variants emerging in other countries were also selected, even if their prevalence in the U.S was still low. A total of 247 specimens were selected from May 2021 to February 2023 for virus isolation in VeroE6/TMPRSS2 or VeroE6/TMPRSS2/ACE2 cells, and viruses were successfully isolated from a total of 143 (57.9%) specimens, representing a total of 59 unique spike protein sequences (Figure 2). To understand the impact of spike mutations on the antigenicity of the virus, isolated viruses were grouped together based on their spike protein sequences and viruses with identical spike mutations were analyzed together. Due to the number of the specimens received and the outcome of the virus isolation, all groups contained various sample sizes.

### 3.2. Neutralization of SARS-CoV-2 Delta, Mu and Omicron Variants by Pooled Monovalent Vaccinee Sera Post the Primary Series

**Figure 3:**
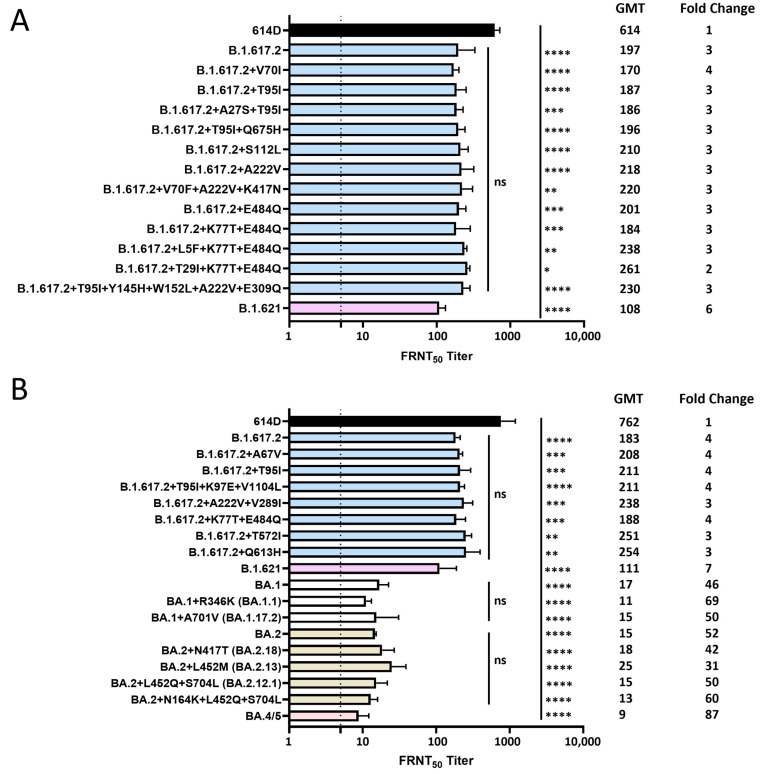
Neutralization of SARS-CoV-2 Delta, Mu and Omicron variants by pooled monovalent vaccinee sera post the primary series. (**A**) A Moderna post-primary series pool and a Pfizer post-primary series pool were each generated from 10 volunteers (no prior SARS-CoV-2 infection) who received two doses of the original mRNA monovalent vaccine from either Moderna or Pfizer-BioNTech. Each isolated SARS-CoV-2 Delta or Mu variant was analyzed against both sera pools using the focus reduction neutralization test (FRNT), and the average FRNT_50_ value was used to represent the neutralization titer of that virus against post-primary series sera pool. (**B**) Post-primary series sera pool with medium range spike-specific antibody titers (1339–3541 BAU/mL) was generated from another five volunteers (no prior SARS-CoV-2 infection) who received two doses of the original mRNA monovalent vaccine (Moderna or Pfizer-BioNTech). This post-primary series medium sera pool was analyzed against various SARS-CoV-2 Delta, Mu and Omicron variants with additional spike mutations. Bars represent geometric mean neutralization titer (GMT) with geometric SD from 2–10 independent repeats of virus with the same spike protein sequence. Significance relative to 614D, B.1.617.2, BA.1 or BA.2 was determined by one-way ANOVA with Dunnett correction on log transformed neutralization titers. *p* values are displayed as * *p* ≤ 0.05, ** *p* ≤ 0.01, *** *p* ≤ 0.001, **** *p* ≤ 0.0001 and not significant (ns) *p* > 0.05. GMT and fold changes compared to 614D are displayed on the side. The dashed line represents the limit of detection at FRNT_50_ of 5.

A Moderna post-primary series pool and a Pfizer post-primary series pool were each generated from 10 volunteers (no prior SARS-CoV-2 infection) who received two doses of the original mRNA monovalent vaccine from either Moderna or Pfizer-BioNTech (Supplementary Table S1). Each isolated SARS-CoV-2 Delta or Mu variant was analyzed against both sera pools using the focus reduction neutralization test (FRNT), and the average FRNT_50_ value was used to represent the neutralization titer of that virus against post-primary series sera pool (Figure 3A). Compared to the 614D reference virus (USA-WA1/2020), B.1.617.2 (Delta) variant had a 3-fold reduction in neutralization titer. All the B.1.617.2 subvariants with additional spike substitutions also showed similar statistically significant reductions in neutralization titer (2–4 fold) compared to the 614D virus, but no significant difference was observed when compared to the ancestral B.1.617.2 variant, suggesting that the additional spike substitutions at the NTD and/or RBD did not have additional impact on the neutralization escape of the B.1.617.2 variant (Figure 3A). The B.1.621 (Mu) variant, which carries the R346K, E484K and N501Y substitutions in the spike RBD domain, showed a 6-fold reduction in neutralization titer compared to the 614D reference (Figure 3A). 

A post-primary series sera pool with medium range spike-specific antibody titers was generated from another five volunteers (no prior SARS-CoV-2 infection) who received two doses of the original mRNA monovalent vaccine (Moderna or Pfizer-BioNTech) (Supplementary Table S1). The capacity of this medium-range post-vaccination sera pool to neutralize various SARS-CoV-2 Delta, Mu and Omicron variants with additional spike substitutions was analyzed. Like the previous analysis using post-primary series sera pool, a 4-fold reduction in neutralization titer compared to the 614D reference virus was observed for the B.1.617.2 variant, but all the Delta variants with additional spike substitutions resulted in comparable serum neutralization titers to the ancestral B.1.617.2 variant, whereas a 7-fold reduction in neutralization titer was observed for the B.1.621 variant (Figure 3B). All the Omicron variants analyzed showed the greatest escape from neutralizing antibodies as illustrated by more than 30-fold reductions in neutralization titers compared to the 614D reference virus, with the BA.4/5 spike resulting in the largest reduction (87-fold). BA.1 subvariants with additional spike substitutions yielded comparable serum neutralization titers to the ancestral BA.1 virus. No significant difference in neutralization titers were observed for BA.2 subvariants when compared to the ancestral BA.2 virus (Figure 3B). 

### 3.3. Neutralization of SARS-CoV-2 Omicron Variants by Individual Human Sera Collected from Post-Third-Dose Monovalent mRNA Vaccine Recipients

**Figure 4:**
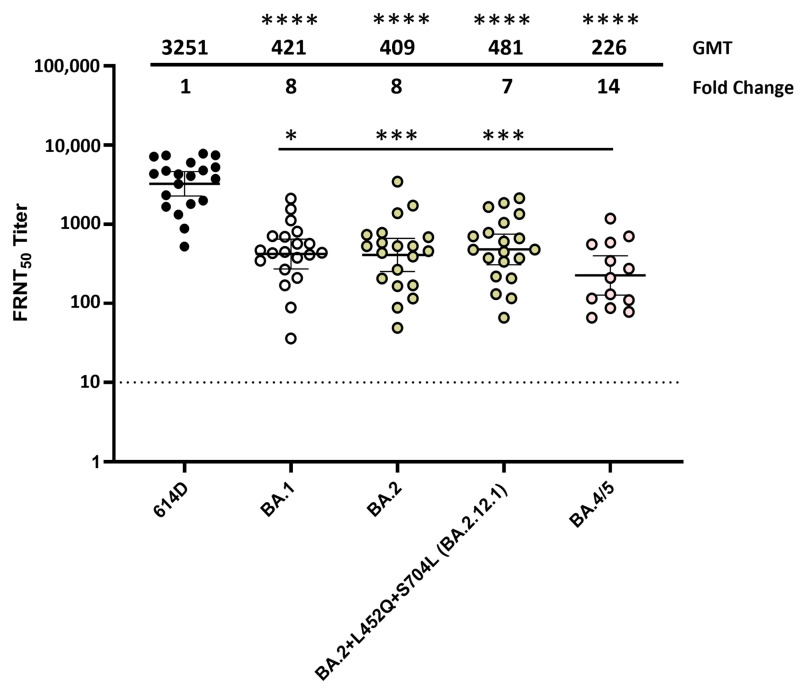
Neutralization of SARS-CoV-2 Omicron variants by individual human sera collected from post-third-dose monovalent mRNA vaccine recipients. Individual sera (each represented by a circle) collected from volunteers (no prior SARS-CoV-2 infection) who received three doses of the original mRNA monovalent vaccine (Moderna or Pfizer-BioNTech) were analyzed against SARS-CoV-2 614D virus (N = 20) and Omicron variants BA.1 (N = 20), BA.2 (N = 20), BA.2.12.1 (N = 20) and BA.4/5 (N = 13) using the FRNT assay. All viruses were isolated from clinical specimens. Bars represent GMT with 95% confidence intervals. Significance relative to 614D or BA.4/5 was determined by one-way repeated measures ANOVA with Dunnett correction on log transformed neutralization titers. *p* values are displayed as * *p* ≤ 0.05, *** *p* ≤ 0.001, **** *p* ≤ 0.0001 and not significant (ns) *p* > 0.05. GMT and fold change compared to the 614D reference virus are displayed at the top of the plots. The dashed line represents the limit of detection at FRNT_50_ of 10.

When using the post-primary series sera pool, serum neutralization titers against all the Omicron lineage variants were very low and approaching the limit of detection. To better characterize the antigenic changes in spike protein that are important in escape from human antibodies, various Omicron variants were analyzed using 13–20 post-third-dose serum samples from volunteers (no prior SARS-CoV-2 infection) who received three doses of the original mRNA monovalent vaccine (Moderna or Pfizer-BioNTech) (Supplementary Table S2). Compared to the post primary series vaccinee sera, post-third-dose vaccinee sera exhibited 25–32 fold increases in neutralization titers against all the Omicron variants analyzed (Figure 4). Compared to the 614D reference virus, BA.1, BA.2 and BA.2.12.1 all resulted in 7–8 fold reductions in neutralization titers, whereas BA.4/5 (which had NTD deletion DEL69/70 and RBD L452R and F486V substitutions compared to BA.2) yielded a 14-fold reduction (Figure 4). No significant difference in neutralization titer was observed among the BA.1, BA.2 and BA.2.12.1 variants; they were all significantly neutralized more easily than the BA.4/5 variant (Figure 4). These data suggest that although three doses of the monovalent mRNA vaccine provided much better protection against the Omicron variants compared to the primary series alone, the amino acid changes that the Omicron lineage evolved resulted in escape from neutralizing antibodies, and the BA.5 showed the largest escape from neutralization. 

### 3.4. Neutralization of SARS-CoV-2 Omicron Variants by Pooled Human Sera Collected from Post-Third-Dose Monovalent mRNA Vaccine Recipients

**Figure 5:**
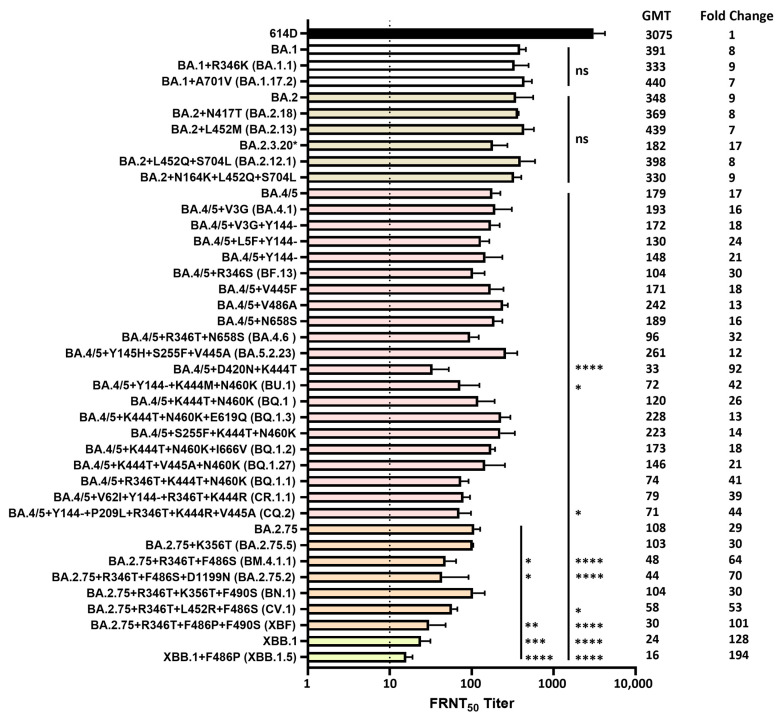
Neutralization of SARS-CoV-2 Omicron variants by pooled human sera from post-third-dose monovalent mRNA vaccine recipients. Post-third-dose sera pool with medium range spike-specific antibody titers (159–3015 BAU/mL) was generated from five volunteers (no prior SARS-CoV-2 infection) who received three doses of the original mRNA monovalent vaccine (Moderna or Pfizer-BioNTech). Various SARS-CoV-2 Omicron variants with additional spike mutations were analyzed using this post-third-dose medium sera pool. All viruses were isolated from clinical specimens. Bars represent GMT with geometric SD from 2–6 independent repeats of virus with the same spike protein sequence. Significance relative to 614D, BA.1, BA.2, BA.4/5 or BA.2.75 was determined by one-way ANOVA with Dunnett correction on log transformed neutralization titers. *p* values are displayed as * *p* ≤ 0.05, ** *p* ≤ 0.01, *** *p* ≤ 0.001, **** *p* ≤ 0.0001 and not significant (ns) *p* > 0.05. Significance to 614D was not displayed as all Omicron variants analyzed had **** *p* ≤ 0.0001. GMT and fold changes compared to 614D are displayed on the side. The dashed line represents the limit of detection at FRNT_50_ of 10. * BA.2.3.20 = BA.2 + M153T + N164K + H245N + G257D + K444R + N450D + L452M + N460K + A484R + R493Q.

Among the 20 individual post-third-dose monovalent mRNA vaccinee sera, 5 sera with medium range spike-specific antibody titers were pooled together as a median range post-third-dose sera pool (Supplementary Table S1) and used to analyze SARS-CoV-2 Omicron variants BA.1, BA.2, BA.2.12.1 and BA.4/5 viruses. For each Omicron variant analyzed, the neutralization titers and fold reductions to the 614D reference virus were very similar to those obtained previously against the 13–20 individual post-third-dose sera (Figure 5), suggesting that the post-third-dose medium sera pool could be used to efficiently characterize the antigenicity of emerging Omicron variants. 

Omicron lineage subvariants isolated from the clinical specimens, which represented a total of 40 unique spike protein sequences, were antigenically characterized using the post-third-dose medium sera pool. The results were grouped based on the ancestral Omicron variants as well as the unique additional spike substitutions. All the Omicron variants showed dramatic escape from neutralizing antibodies, illustrated by reductions in neutralization titers compared to the 614D reference virus (*p* < 0.0001) (Figure 5). No significant differences in neutralization titers were observed when comparing the BA.1 or BA.2 subvariants with additional spike substitutions to the ancestral BA.1 or BA.2 virus. BA.2.3.20, which contains additional spike substitutions M153T, N164K, H245N, G257D, K444R, N450D, L452M, N460K and E484R, resulted in the lowest neutralization titer among all the BA.1 and BA.2 subvariants analyzed, which was 17-fold reduced compared to the 614D reference virus (*p* < 0.0001) (Figure 5). Among the BA.4/5 subvariants with additional spike substitutions, the substitution and/or deletion in the spike NTD alone (V3G, L5F, Y144-) did not have a significant impact on the neutralization titer compared to the ancestral BA.4/5 virus; neither did the substitutions in the RBD and the S1 subdomain alone except for the subvariant with K444T + D420N substitutions in the RBD. This subvariant exhibited 5.4-fold further reduction in serum neutralization titers compared to the ancestral BA.4/5 virus (*p* < 0.0001) and 92-fold reduction compared to the 614D reference virus (*p* < 0.0001) (Figure 5). Subvariant BQ.1.1, with R346T + K444T + N460K substitutions in the RBD, also resulted in low neutralization titers, which were 41-fold reduced compared to the 614D reference virus (*p* < 0.0001).

Interestingly, subvariants with both the NTD deletion Y144- and RBD substitutions, such as BU.1 (Y144- + K444M + N460K), CR.1.1 (V62I + Y144- + R346T + K444R) and CQ.2 (Y144- + P209L + R346T + K444R + V445A), resulted in 2.3–2.5-fold further reductions in neutralization titers compared to the ancestral BA.4/5 virus (*p* < 0.05 for BU.1 and CQ.2) and 39–44-fold reductions compared to the 614D reference virus (*p* < 0.0001). However, subvariants with both RBD substitutions and other NTD substitutions, such as Y145H + S255F + V445A and S255F + K444T + N460K, did not yield reduced neutralization titers compared to the ancestral BA.4/5 virus (Figure 5).

The BA.2.75 variant resulted in a 29-fold reduction in neutralization titer compared to the 614D reference virus (*p* < 0.0001). Among the BA.2.75 subvariants with additional spike substitutions, a 1.9–2.5-fold further reduction in neutralization titers compared to the ancestral BA.2.75 was observed in subvariants with additional R346T + F486S substitutions, including BM.4.1.1 (R346T + F486S, *p* < 0.05), BA.2.75.2 (R346T + F486S + D1199N, *p* < 0.05) and CV.1 (R346T + L452R + F486S), which resulted in 53–70-fold reductions compared to the 614D reference virus (*p* < 0.0001) (Figure 5). 

XBF was a recombinant between Omicron subvariants BA.5.2.3 and BA.2.75.3, which contains additional spike substitutions R346T, F486P and F490S compared to BA.2.75. XBF yielded a 3.6-fold further reduction in neutralization titer compared to BA.2.75 (*p* < 0.01), which resulted in a 101-fold reduction compared to the 614D reference virus (*p* < 0.0001) (Figure 5). XBB.1 was a recombinant between the Omicron subvariants BA.2.10.1 and BA.2.75 that gave rise to the next predominant Omicron lineage. Compared to BA.2.75, the XBB.1 spike protein not only has R346T, L368I, V445P, F486S and F490S in the RBD, but also contains different substitutions/deletions at the NTD, such as V83A, Y144-, H146Q, Q183E, V213E and G252V. XBB.1 yielded a 4.5-fold further reduction in neutralization titer compared to BA.2.75 (*p* < 0.001) and a 128-fold reduction compared to the 614D reference virus (*p* < 0.0001) (Figure 5). Compared to XBB.1, XBB.1.5 differs by only one amino acid. It has the F486P substitution (instead of F486S), which resulted in 6.8-fold further reduction compared to BA.2.75 (*p* < 0.0001). Among the Omicron subvariants analyzed, XBB.1.5 also exhibited the largest reduction in neutralization titers (194-fold) compared to the 614D reference virus (*p* < 0.0001) (Figure 5). BM.4.1.1, BA.2.75.2, CV.1, XBF, XBB.1 and XBB.1.5 also resulted in 3- to 11-fold further reductions in neutralization titers compared to the BA.4/5 virus (*p* < 0.0001, except for CV.1: *p* < 0.05).

### 3.5. Neutralization of SARS-CoV-2 Omicron Variants by Individual Human Sera Collected from Vaccine Recipients Who Received the Bivalent mRNA Booster as the Fourth Dose

**Figure 6:**
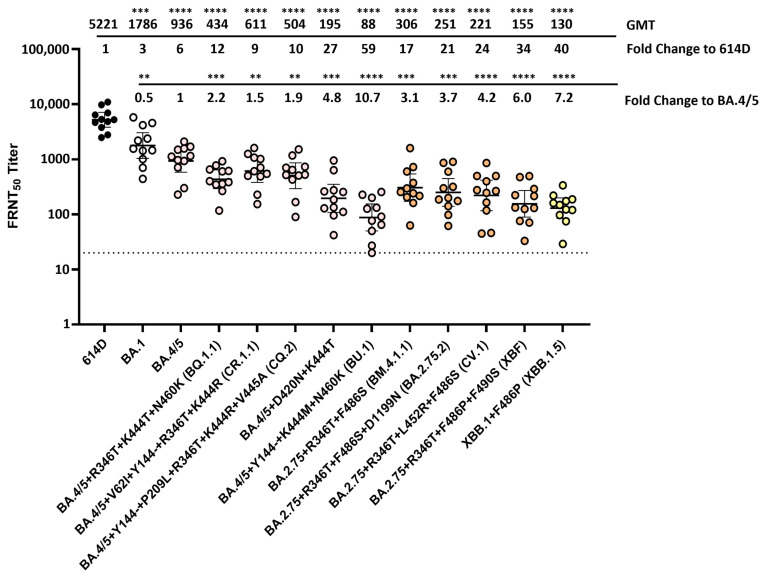
Neutralization of SARS-CoV-2 Omicron variants by individual human sera collected from vaccine recipients (no prior SARS-CoV-2 infection) who received the bivalent mRNA booster as the fourth dose. Eleven human serum samples (each represented by a circle) collected from volunteers who received three doses of monovalent mRNA vaccine and one dose of the bivalent mRNA booster (original SARS-CoV-2 virus + Omicron variant BA.4/5) were analyzed against SARS-CoV-2 614D virus and various Omicron variants using the FRNT assay. All viruses were isolated from clinical specimens. Bars represent GMT with 95% confidence intervals. Significance relative to 614D or BA.4/5 was determined by one-way repeated measures ANOVA with Dunnett correction on log transformed neutralization titers. *p* values are displayed as ** *p* ≤ 0.01, *** *p* ≤ 0.001, **** *p* ≤ 0.0001 and not significant (ns) *p* > 0.05. GMT and fold change compared to 614D or BA.4/5 are displayed at the top of the plots. The dashed line represents the limit of detection at FRNT_50_ of 20.

Among the BA.4/5 subvariants, BA.2.75 subvariants and the recombinant Omicron subvariants, ten resulted in more than 35-fold reductions in serum neutralization titers compared to the 614D reference when analyzed against the post-third-dose medium sera pool. Those ten subvariants were selected to analyze against 11 individual human sera collected from vaccinees (no prior SARS-CoV-2 infection) who received three doses of monovalent mRNA vaccine and one dose of the bivalent mRNA booster (Supplementary Table S3). Compared to the post-third-dose monovalent mRNA vaccinee sera, post-bivalent sera exhibited 4- to 8-fold increases in neutralization titers against all the Omicron variants analyzed, except for the BU.1 variant, which had the Y144- deletion in the spike NTD plus K444M and N460K substitutions in the RBD (Figure 6). Compared to the 614D reference virus, BA.4/5 subvariants BQ.1.1, CR.1.1 and CQ.2 yielded 9–12-fold reductions in neutralization titers when analyzed using the post-bivalent vaccinee sera (*p* < 0.0001), which was less than the 39- to 44-fold reduction against the post-third-dose monovalent sera (Figure 6). Compared to the 614D virus, the BA.4/5 subvariant with D420N and K444T substitutions in the RBD exhibited 27-fold reductions in neutralization titers when analyzed against the post-bivalent sera (*p* < 0.0001), which was also less than the 92-fold reduction against the post-third-dose monovalent sera. Interestingly, despite resulting in a higher neutralization titer, subvariant BU.1 still had a 59-fold reduction compared to the 614D virus when analyzed using the post-bivalent sera (*p* < 0.0001) (Figure 6). Compared to the ancestral BA.4/5 virus, all the BA.4/5 subvariants analyzed had significant reductions (1.5- to 10.7-fold) in neutralization titers when analyzed against the post-bivalent sera. The fold reduction was only slightly less than that against the post-third-dose monovalent sera (Figure 2).

Compared to the 614D reference virus, BA.2.75 subvariants BM.4.1.1, BA.2.75.2 and CV.1 resulted in 17- to 24-fold reductions in neutralization titers when analyzed against the post-bivalent sera (*p* < 0.0001). This was less than the 53- to 70-fold reductions against the post-third-dose monovalent sera (Figure 6). The recombinant Omicron subvariant XBF, which had the F486P instead of F486S substitution, exhibited a 34-fold reduction in neutralization titer compared to 614D when analyzed against the post-bivalent sera (*p* < 0.0001), which was less than the 101-fold reduction against the post-third-dose monovalent sera (Figure 6). The recombinant Omicron subvariant XBB.1.5, which also had the F486P substitution plus additional substitutions/deletions at the NTD, exhibited the largest fold reduction (40-fold) in neutralization titer compared to 614D when analyzed against the post-bivalent sera (*p* < 0.0001). This was also less than the 194-fold reduction obtained against the post-third-dose monovalent sera (Figure 6). Compared to the BA.4/5 virus, all the BA.2.75 subvariants and recombinant subvariants analyzed resulted in significant reductions (3.1 to 7.2-fold) in neutralization titers when analyzed against the post-bivalent sera. The fold reduction was similar or slightly less than that against the post-third-dose monovalent sera (Figure 2).

Overall, these data showed that an additional bivalent vaccination targeting both the original SARS-CoV-2 spike and Omicron BA.4/5 spike increased the level of neutralizing antibodies against a wide range of emerging Omicron lineage variants. However, the further-evolved BU.1, XBF and XBB.1.5 variants still showed large reductions in antibody neutralization (34- to 59-fold) when compared to the 614D reference virus.

## 4. Discussion

This study represents systematic antigenic characterization of many SARS-CoV-2 variants from the Delta, Mu and Omicron lineages using panels of vaccinee sera generated throughout the first three years of the COVID-19 pandemic. All the Delta subvariants analyzed against the post-primary series vaccinee sera resulted in similar serum neutralization reductions (2–4 fold) compared to the 614D reference virus, which is concordant with other published studies (Figure 2) [22,24,25]. Although BA.1 and BA.2 subvariants exhibited high levels of neutralizing antibody escape (30- to 70-fold) compared to the 614D virus when analyzed using post-primary series vaccinee sera, post-third-dose original monovalent vaccinee sera provided better protection against those subvariants with higher neutralization titers and lower fold reductions (7- to 17-fold). However, the continued evolution of the virus resulted in more antigenic drift and escape from neutralizing antibodies. This is illustrated by BA.4/5 subvariants BU.1, BQ.1.1, CR.1.1, CQ.2 and BA.4/5 + D420N + K444T, subsequent BA.2.75 subvariants BM.4.1.1, BA.2.75.2 and CV.1 and subsequent recombinant variants XBF, XBB.1 and XBB.1.5, which each resulted in increasing escape from neutralizing antibodies (39- to 194-fold) when analyzed using sera post third-dose of the original monovalent vaccine (Figure 2). The updated original and BA.4/5 bivalent vaccine resulted in sera that provided better neutralization of the BA.4/5, BA.2.75 and XBB lineage subvariants (9- to 59-fold reductions) compared to the 614D virus. Nevertheless, the accumulation of amino acid substitutions resulted in increasing neutralization escape, especially in the recombinant Omicron variants and the BA.4/5 subvariants with both the NTD deletion and RBD substitutions K444M + N460K (Figure 2). 

A previous study using the high-throughput deep mutational scanning predicted that residues R346, K444–G446, N450, K356, N417, L455, N460 and A484 would be the RBD mutation hotspots for BA.4/5 subvariants [26]. In this study, we successfully isolated and characterized BA.4/5 subvariants with additional spike substitutions at residues R346, D420, K444–G446, N460 and F486 in the RBD and at various locations in the NTD. We found that among all the BA.4/5 subvariants analyzed, subvariants BU.1, which had both NTD deletion Y144- and RBD substitutions K444M + N460K, resulted in the most substantial serum neutralization resistance and remained highly evasive even against post-fourth dose bivalent vaccinee sera (Figure 2). Another study using pseudovirus also showed that BA.4/5 subvariants with Y144-, K444X and N460K, such as BQ.1.1.10 (Y144- + R346T + K444T + N460K) and BA.4.6.3 (Y144- + R346T + K444N + N460K + N658S), were among the most antibody-evasive viruses analyzed against plasma from individuals who had been infected with BA.5 after receiving three doses of CoronaVac [26]. N460K and K444T were shown to be the main driving force for the enhanced antibody escape of BA.4/5 subvariants BQ.1 and BQ.1.1 [27]. The N460K substitution was shown to confer resistance to RBD class 1 antibodies, while the K444T substitution was shown to interfere with RBD class 3 antibody recognition [27,28]. Residue Y144 is in the NTD antigenic supersite and the Y144 deletion conferred resistance to the NTD class α antibodies [20,29]. Although BU.1 is highly antibody-evasive, it never exceeded 1% prevalence in any given week in the U.S., suggesting that it provides less fitness advantage compared to other circulating variants. BA.4/5 subvariants with RBD substitutions D420N and K444T also exhibited substantial neutralization resistance to both post-third dose monovalent and post-fourth dose bivalent vaccinee sera (Figure 2). RBD class 1 and 4 antibodies are sensitive to substitutions at the D420 residue, and class 3 antibodies are sensitive to the K444T substitution [17,27,30]. Another study has shown that a pseudovirus with R346W, D420N and K444N substitutions in the BA.5 spike protein was resistant to 38 of 40 broadly neutralizing antibodies tested [30]. 

RBD substitutions R346T/S, K356T, N417Y/H/I/T, K444E/Q/N/T/M, V445D/G/A, N450T/D/K/S, L452R, I468N, A484P, F486S/V and F490S/Y were predicted to emerge in BA.2.75 subvariants as escape mutations [26]. Here, we characterized BA.2.75 subvariants with additional spike substitutions, such as R346T, K356T, L452R, F486S/P, F490S and D1199N, among which BA.2.75 subvariants with both R346T and F486S/P substitutions resulted in greater neutralization escape compared to the parental BA.2.75 variant (Figure 2). This observation is concordant with another study showing that when testing R346T, F486S and D1199N as single or double substitutions using pseudovirus with a BA.2.75 spike construct, R346T and F486S exhibited a synergistic effect when combined and were the major drivers of enhanced neutralization escape from both post-third dose vaccinee sera and BA.4/5 convalescent sera [27]. F486 is located within the receptor binding motif (RBM), and the F486S substitution negatively impacts the recognition by the RBD class 1 and 2 monoclonal antibodies, which is concordant with another study showing that RBD group B neutralizing antibodies are very sensitive to changes at the F486, N487 and G476 sites [17,27]. R346T is a well-known escape substitution, which is located outside of the RBM and exhibited evasion of RBD class 3 or group E neutralizing antibodies [26,27,31]. Recombinant variants between the BA.2.10.1 and BA.2.75, XBB.1 and XBB.1.5, not only have R346T and F486S/P substitutions in their spike RBD but also contain additional substitutions and deletions in the NTD, such as V83A, Y144-, H146Q and G252V. The NTD class α antibodies are sensitive to Y144 deletion and H146 substitution, whereas the NTD class δ antibodies are affected by V83A [20,26]. The V213X harbored by all the Omicron subvariants could escape the NTD class γ antibodies [19]. Altogether, those NTD deletions and substitutions would enable XBB.1 and XBB.1.5 to escape most of the NTD-targeting antibodies, which may explain their further neutralization escape compared to the BA.2.75 subvariants. Even with the post-bivalent vaccinee sera, BA.2.75 subvariants with R346T and F486S/P substitutions and the recombinant variants still exhibited substantial neutralization escape, which is consistent with other studies with bivalent vaccines (Figure 2) [32,33,34]. 

This study clearly highlights the specific amino acid changes and epitopes that had larger impacts on escape from neutralization by human antibodies and provides an explanation as to why certain lineages (e.g., BA.1, BA.5, XBB.1.5) had a fitness advantage in humans and rather rapidly displaced their progenitors. For example, the F486P substitution that differentiates XBB.1 and XBB.1.5 had a large impact on neutralization escape, resulted in more rapid growth of XBB.1.5. In June 2023, the FDA updated the 2023–2024 formulation of the COVID-19 vaccines for use in the U.S. with a monovalent XBB.1.5 composition. The updated COVID-19 vaccines were authorized for emergency use by the FDA and recommended for eligible populations by the CDC in September 2023 [35]. As of September 2023, the variant proportion of XBB.1.5 was decreasing whereas the proportion of XBB.1.16 and XBB.1.9 subvariants was increasing in the U.S. (Figure 1). XBB.1.9 subvariants, such as XBB.1.9.1 and XBB.1.9.2, had the same spike amino acid profile as XBB.1.5. XBB.1.9.2 descendent lineage EG.5 had an additional F456L substitution in the spike protein compared to XBB.1.5. Studies have shown that compared to XBB.1.5, EG.5 subvariants with the F456L substitution exhibited increased resistance to neutralization when analyzed against post-bivalent sera [36,37]. XBB.1.16 had two substitutions, E180V and T478R, in the spike protein compared to XBB.1.5, but exhibited similar levels of neutralization resistance to XBB.1.5 when analyzed against BA.5 breakthrough infection sera [38]. Currently, variant JN.1, which is a descendent of BA.2.86, is the predominant variant circulating in the U.S. and had more than 30 amino acid substitutions in the spike protein compared to XBB.1.5. Furthermore, F486P also arose via parallel evolution in the BA.2.86/JN.1 lineage, illustrating that this change confers a selective advantage in the human population. Studies have shown that JN.1 was highly resistant to neutralization when analyzed against the post-bivalent sera, but post-monovalent XBB.1.5 vaccine sera provided much-enhanced neutralization against the JN.1 variant [39,40,41]. 

Limitations of this study include the following: (1) serum samples from a relatively small number of vaccine recipients were analyzed; (2) these individuals may not have been representative of others; (3) when using the pooled sera, individual-person variation from the pool mean could not be assessed.

## 5. Conclusions

Clearly, SARS-CoV-2 has the evolutionary power to continue to evolve to escape immune pressures, and it will continue to lead to further antigenic drift. Therefore, it is important to keep conducting evolutionary analysis, selecting emerging variants for further characterization and to determine their potential as new vaccine antigens. This study provides the framework for long-term genetic surveillance and antigenic characterization of emerging SARS-CoV-2 variants to evaluate their potential impact on the effectiveness of current vaccines. Identification and evaluation of potential new vaccine antigens is critical to a timely public health decision on future COVID-19 vaccines. 

## Data Availability

The data presented in the study are included in the article and supplementary material, further inquiries can be directed to the corresponding author.

## Supplementary Materials

The following supporting information can be downloaded at: https://www.mdpi.com/article/10.3390/vaccines12050505/s1, Table S1: Vaccinee sera pools used in this study; Table S2: Individual human sera collected from vaccine recipients who received 3 doses of the original mRNA monovalent vaccine; Table S3: Individual human sera collected from vaccine recipients who received 3 doses of the original monovalent mRNA vaccine and 1 dose of the bivalent mRNA booster.
