# Supplementary material for: Antigenic Characterization of Circulating and Emerging SARS-CoV-2 Variants in the U.S. throughout the Delta to Omicron Waves
Source: Vaccines (Basel). 2024 May 7;12(5):505. doi: 10.3390/vaccines12050505 (PMC11125585; doi:10.3390/vaccines12050505)
Supplement: Supplementary file 1 [file vaccines-12-00505-s001.zip › vaccines-2967716-supplementary.pdf]

Supplementary Table S1. Vaccinee sera pools used in this study.

	**Moderna post-primary series pool**	**Pfizer post-primary series pool**	**Medium range post-primary series pool**	**Medium range post 3rd-dose pool**
**Number of serum samples**	10	10	5	5
**Age in years, median (range)**	23 (19-64)	32.5 (21-54)	49 (25-55)	52 (28-70)
**Sex assigned at birth**	Male (5)/Female (5)	Male (5)/Female (5)	Male (4)/Female (1)	Male (2)/Female (3)
**Days between sampling and last vaccination, median (range)**	32 (16-42)	31 (16-39)	28 (15-42)	34 (14-36)
**Vaccine Type**	Moderna, monovalent	Pfizer-BioNTech, monovalent	Moderna or Pfizer-BioNTech, monovalent	Moderna or Pfizer-BioNTech, monovalent
**Vaccine dose**	Two doses	Two doses	Two doses	Three doses
**Anti-SARS-CoV-2 Spike IgG [1] (BAU/mL), median (range)**	2805 (605-8066)	2398 (440-5227)	2973 (1339-3541)	2069 (159-3015)
**Prior SARS-CoV-2 infection [2]**	No	No	No	No

[1] Anti-SARS-CoV-2 Spike IgG titers were determined against ancestral SARS-CoV-2.

[2] No prior SARS-CoV-2 infection was based on negative anti-nucleocapsid antibodies detected in the serum samples.

Supplementary Table S2. Individual human sera collected from vaccine recipients who received 3 doses of the original mRNA monovalent vaccine.

Sample ID	Age	Sex (assigned at birth)	Vaccine product	Date of 3rd vaccination	Serum collection date	Prior COVID[2]	Anti-SARS-CoV-2 Spike IgG (BAU/mL)[1]
3002086116*	25	Male	Moderna	11/5/2021	12/6/2021	No	33
3002086117	33	Female	Pfizer-BioNTech	11/11/2021	12/7/2021	No	63
3002086118	28	Female	Moderna	11/5/2021	12/10/2021	No	70
3002086119*	35	Female	Moderna	10/7/2021	12/2/2021	No	20
3002086120*	48	Male	Moderna	11/5/2021	12/6/2021	No	92
3002086122*	65	Male	Moderna	11/26/2021	12/20/2021	No	22
3002086124*	21	Male	Pfizer-BioNTech	11/16/2021	12/7/2021	No	67
3002086126*	52	Female	Moderna	12/3/2021	12/17/2021	No	159
3002086127*	27	Female	Moderna	11/18/2021	12/6/2021	No	168
3002086135	28	Female	Pfizer-BioNTech	11/19/2021	12/7/2021	No	92
3001241287*	53	Female	Pfizer-BioNTech	10/8/2021	11/2/2021	No	2069
3001241290	70	Male	Pfizer-BioNTech	9/29/2021	11/4/2021	No	1563
3001241286*	52	Male	Pfizer-BioNTech	9/28/2021	10/26/2021	No	6247
3001241291	39	Female	Pfizer-BioNTech	9/29/2021	11/4/2021	No	11641
3001241296	42	Female	Moderna	11/2/2021	12/1/2021	No	6961
3001241292	28	Female	Pfizer-BioNTech	10/19/2021	11/22/2021	No	3015
3001241289*	30	Male	Pfizer-BioNTech	9/30/2021	11/4/2021	No	2314
3001241307*	73	Male	Pfizer-BioNTech	10/29/2021	11/22/2021	No	13457
3001241335*	46	Male	Pfizer-BioNTech	11/4/2021	12/2/2021	No	9277
3001241342*	23	Male	Moderna	11/5/2021	11/22/2021	No	13978

[1] Anti-SARS-CoV-2 Spike IgG titers were determined against ancestral SARS-CoV-2.

[2] No prior COVID was based on negative anti-nucleocapsid antibodies detected in the serum samples.

* 13 out of 20 sera were analyzed against the isolated Omicron variants BA.4/5.

Supplementary Table S3. Individual human sera collected from vaccine recipients who received 3 doses of the original monovalent mRNA vaccine and 1 dose of the bivalent mRNA booster.

Sample ID	Age	Sex (assigned at birth)	Vaccine product	Date of bivalent booster	Serum collection date	Prior COVID[2]	Anti-SARS-CoV-2 Spike IgG (BAU/mL)[1]
3032569196	48	Male	Moderna	9/28/2022	10/14/2022	No	8983
3032569198	36	Male	Pfizer-BioNTech	10/12/2022	10/28/2022	No	6266
3032569201	25	Male	Pfizer-BioNTech	9/28/2022	11/8/2022	No	6403
3032569190	55	Male	Pfizer-BioNTech	9/27/2022	10/13/2022	No	6797
3032569184	39	Female	Pfizer-BioNTech	9/30/2022	11/10/2022	No	3086
3032569192	63	Female	Moderna	9/28/2022	10/19/2022	No	8040
3032569188	48	Female	Pfizer-BioNTech	10/11/2022	10/26/2022	No	11840
3003753004	49	Female	Moderna	11/8/2022	12/1/2022	No	13577
3003753064	61	Female	Pfizer-BioNTech	10/31/2022	11/17/2022	No	1775
3003753093	42	Female	Pfizer-BioNTech	11/9/2022	11/30/2022	No	3298
3003753048	47	Male	Moderna	11/23/2022	12/5/2022	No	6078

[1] Anti-SARS-CoV-2 Spike IgG titers were determined against ancestral SARS-CoV-2.

[2] No prior COVID was based on negative anti-nucleocapsid antibodies detected in the serum samples.
